# Anisotropic diamond etching through thermochemical reaction between Ni and diamond in high-temperature water vapour

**DOI:** 10.1038/s41598-018-25193-2

**Published:** 2018-04-27

**Authors:** Masatsugu Nagai, Kazuhiro Nakanishi, Hiraku Takahashi, Hiromitsu Kato, Toshiharu Makino, Satoshi Yamasaki, Tsubasa Matsumoto, Takao Inokuma, Norio Tokuda

**Affiliations:** 1 Graduate School of Natural Science and Technology, Kanazawa University, Kanazawa Ishikawa, 920-1192 Japan; 2 National Institute of Advanced Industrial Science and Technology, Tsukuba Ibaraki, 305-8568 Japan

## Abstract

Diamond possesses excellent physical and electronic properties, and thus various applications that use diamond are under development. Additionally, the control of diamond geometry by etching technique is essential for such applications. However, conventional wet processes used for etching other materials are ineffective for diamond. Moreover, plasma processes currently employed for diamond etching are not selective, and plasma-induced damage to diamond deteriorates the device-performances. Here, we report a non-plasma etching process for single crystal diamond using thermochemical reaction between Ni and diamond in high-temperature water vapour. Diamond under Ni films was selectively etched, with no etching at other locations. A diamond-etching rate of approximately 8.7 μm/min (1000 °C) was successfully achieved. To the best of our knowledge, this rate is considerably greater than those reported so far for other diamond-etching processes, including plasma processes. The anisotropy observed for this diamond etching was considerably similar to that observed for Si etching using KOH.

## Introduction

Diamond has attracted considerable attention as a promising material for high-power devices^1–6^, quantum devices^7–10^, cold cathodes^10–13^ and microelectromechanical systems^14–17^ because of its outstanding physical and electrical properties^18^. To fabricate these devices, etching technique to control diamond geometry is imperative. Furthermore, the geometric structures of the etched diamond significantly influence device-performances. For instance, the trench structure, which enables no JFET resistance and a high cannel density, can drastically suppress the on-resistance of power devices compared to the other structures^19^. However, little progress has been made in studies related to diamond-based power devices with trench structures^2^ due to the difficulty involved in shaping diamond trenches associated with two major reasons related to diamond etching. One reason involves the use of anisotropic wet processes, such as KOH processes, which are frequently utilised to form Si trench structures but are not effective for diamond because of its chemical stability. The other reason is that plasma processes currently used for diamond etching exhibit low selectivity (diamond/masks)^20^, and plasma-induced damage to diamond^21,22^ leads to the deterioration in the device’s performance^23^. Hence, it is crucial to develop a highly selective non-plasma process for diamond etching.

In the mechanical machining field, it is well known that diamond tools wear severely when used for workpieces containing transition metals such as Fe, Ni, Co and Ti because of thermochemical reaction between the diamond and the metals^24,25^. This reaction has been employed for etching and patterning diamond^26–30^. For example, V. G. Ralchenko *et al*. reported etching rate as high as 8 µm/min for polycrystalline diamond using the thermochemical reaction between Fe and diamond in high-temperature H_2_^26^. However, their process was not effective for single crystal diamond^26^. On the other hand, Morofushi *et al*. proposed an etching process for single crystal diamond based on the reaction between Ni and diamond in high-temperature air with a rate of ~0.25 µm/min^27^. The rate was comparable to that of plasma processes, whereas significantly lower rates had been reported in etching methods for single crystal diamond using the same reaction in high-temperature non-oxidative gases, such as N_2_^27^, Ar^28^ and H_2_^29,30^. This indicates that the oxidation of Ni by high-temperature O_2_ present in the air is essential for achieving a high etching rate. However, O_2_ oxidises Ni^31^ as well as diamond exposed to the air. Accordingly, diamond is etched away^27^ as CO_2_ or CO gas^32–34^ in their process. Thus, the selectivity (diamond contacted with Ni/diamond uncontacted with Ni) decreases. To simultaneously achieve high selectivity and rate, the selective oxidation of Ni by high-temperature water vapour was targeted^35^. Herein, an innovative etching process for single crystal diamond based on a thermochemical reaction between Ni and diamond in high-temperature water vapour is reported. The process was conducted to diamond (100) and (111) to confirm whether the process is a crystal anisotropic etching as with KOH-Si etching processes, where Si (111) surfaces are flattened because anisotropic Si etching takes place by etch-step traveling along the {111} planes^36^. In addition, the diamond etching mechanism is discussed.

## Results

### Etching of diamond (100) surfaces

Diamond (100) surfaces were etched simply as explained below. Ni films were deposited on single-crystal diamond (100) substrates. Then, the samples were annealed in water vapour. Hereinafter, this annealing is referred to as ‘wet annealing’. After wet annealing at 1000 °C for 3 min, NiO formed at the deposited film surface was detected by X-ray photoelectron spectroscopy (XPS). Judging from the XPS result, the thickness of NiO was presumed to be more than 60 nm. Finally, the deposited films were removed using a hot mixed acid (HMA).

**Figure 1:**
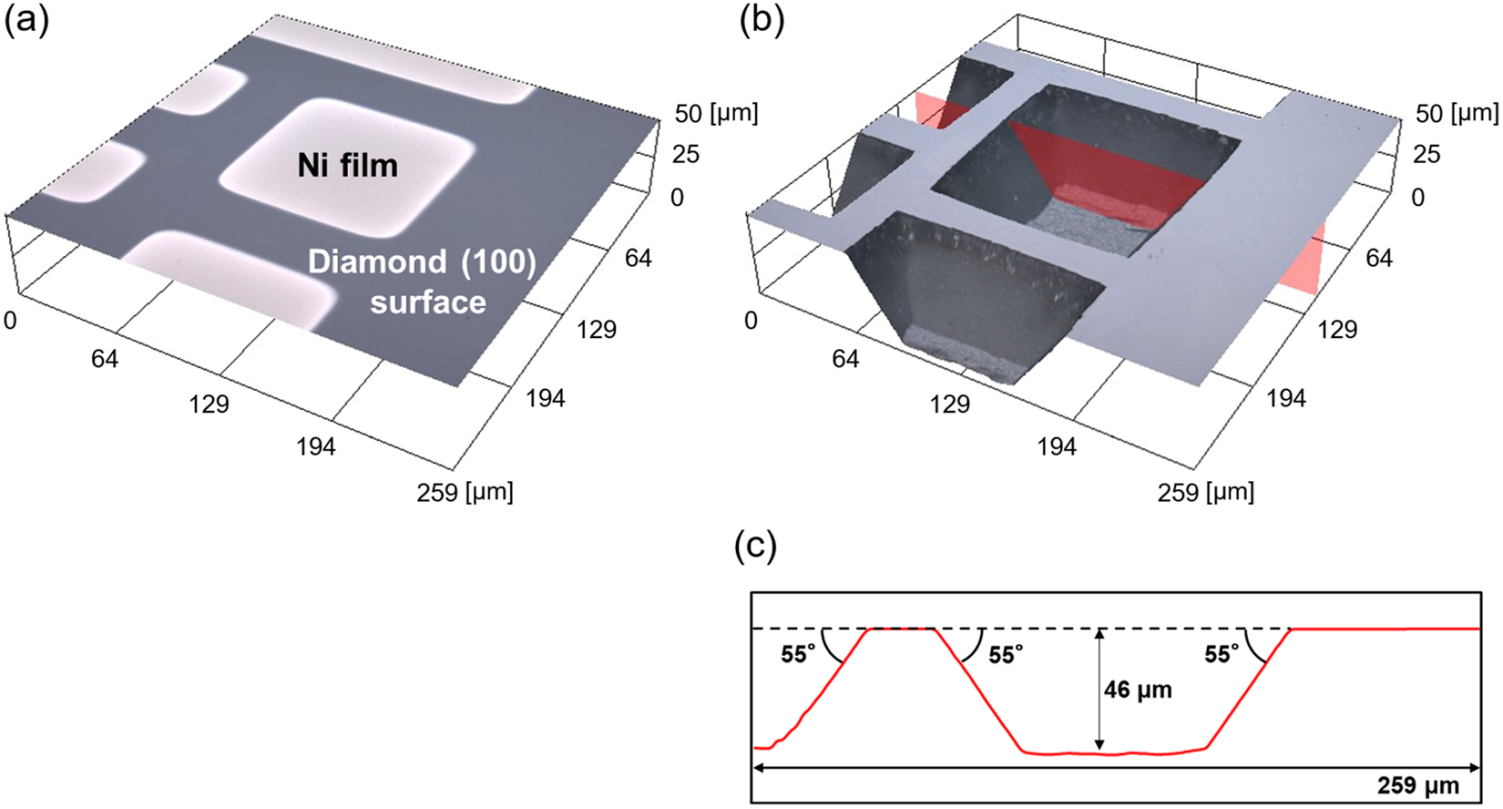
Three-dimensional (3D) laser microscopy (LM) images of the sample morphology (**a**) after depositing Ni films with sizes of 50 × 50, 100 × 100, 200 × 200 µm^2^ and (**b**) after wet annealing at 1000 °C for 3 min and the removal of the deposited films, respectively. (**c**) Cross-sectional image corresponding to the red area of (**b**).

Figure 1 depicts three-dimensional (3D) laser microscopy (LM) images of the sample surface morphology (a) after depositing Ni films with sizes of 50 × 50, 100 × 100, 200 × 200 µm^2^ and (b) after wet annealing at 1000 °C for 3 min and the removal of the deposited films, respectively. Figure 1c is cross-sectional image corresponding to the red area of Fig. 1b. Diamond under the deposited Ni films selectively etched, and diamond trenches were formed. The mean trench depth of 46 µm was obtained by averaging depth of ten trenches.

Meanwhile, according to the LM measurements, the thickness of the Ni-undeposited area of the diamond substrate did not change through this experiment. The diamond trenches were pointed or truncated pyramids (depending on the size of the Ni films and etching depth) surrounded by four side walls. The angle between the side walls and (100) planes was approximately 55°.

**Figure 2:**
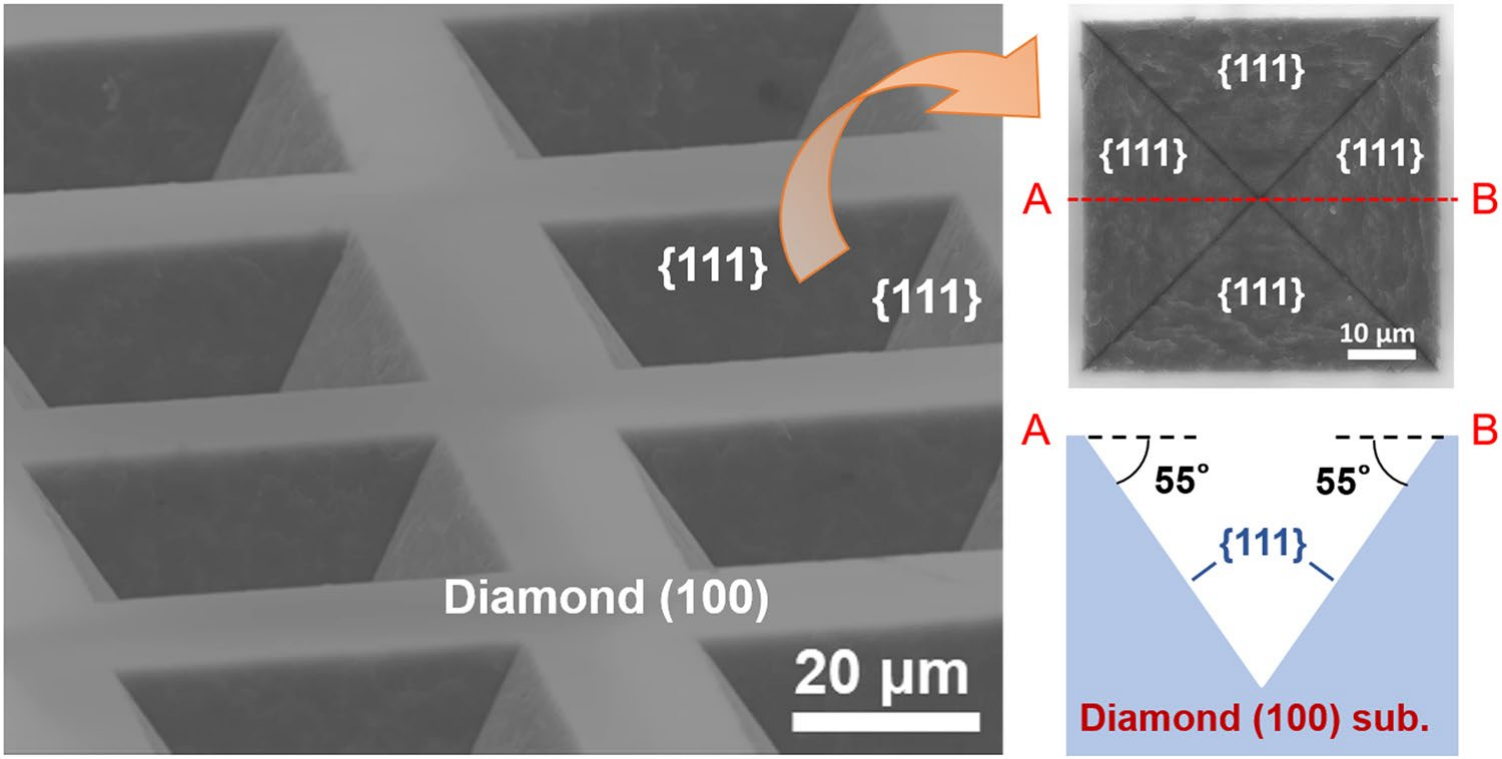
Scanning electron microscopy image (SEM) images of the sample surface morphology and its schematic cross-sectional image after the deposition of an array of Ni films (30 × 30 µm^2^), wet annealing (1000 °C for 3 min) and the removal of the deposited films.

Figure 2 shows the scanning electron microscopy (SEM) images of the sample surface morphology as well as a schematic of its cross-sectional image after the deposition of an Ni film array with a size of 30 × 30 µm^2^, wet annealing at 1000 °C for 3 min and the removal of deposited films. An array of inverted-pyramidal trenches comprising four side walls was observed on the diamond (100) surface.

**Figure 3:**
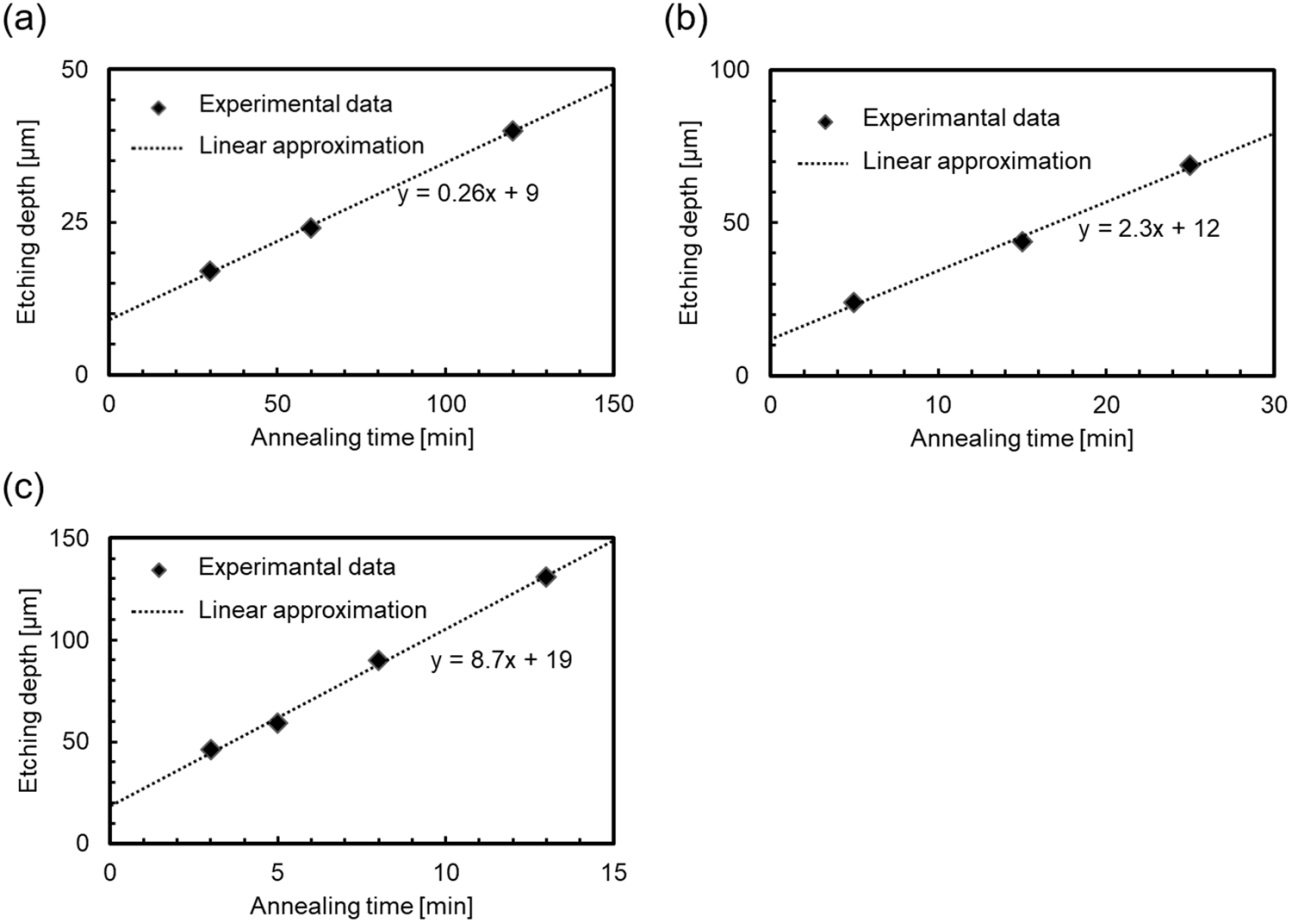
Etching depth as a function of wet-annealing time at (**a**) 900 °C, (**b**) 950 °C and (**c**) 1000 °C with approximation straight lines. The intercepts on the vertical axes (etching depth at 0 min) represent that etching occurred during the temperature raising and lowering processes.

Figure 3 shows the etching depth of diamond as a function of the wet annealing time at (a) 900 °C, (b) 950 °C and (c) 1000 °C. The intercepts on the vertical axes (etching depth at 0 min) represent that etching occurred during the temperature raising and lowering processes. The etching depth linearly increased with the wet annealing time at all the temperatures. From the inclinations of the approximation straight lines, the etching rates at 900, 950 and 1000 °C were estimated to be approximately 0.26, 2.3 and 8.7 µm/min, respectively.

**Figure 4:**
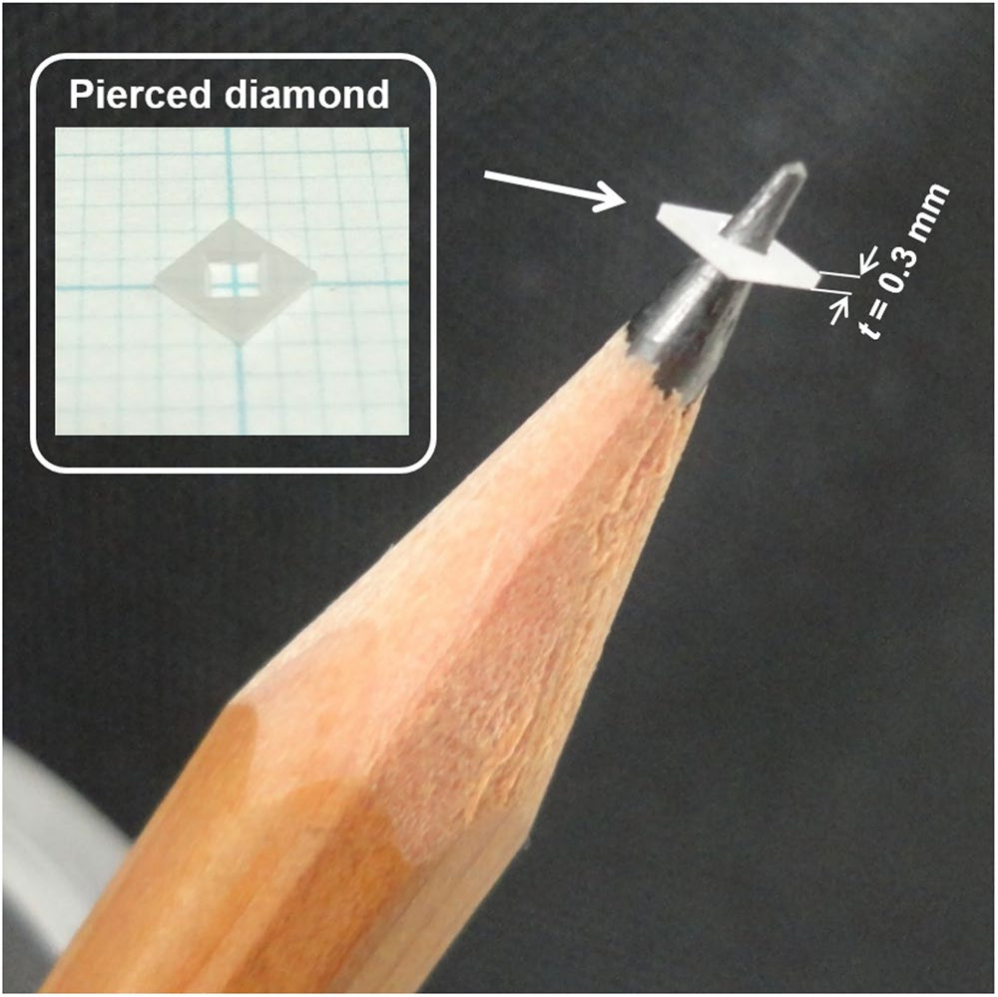
Photographs of the diamond (100) substrate (thickness is approximately 0.3 mm) completely pierced by the proposed process with wet annealing at 1000 °C for 40 min.

Figure 4 shows photographs of the diamond (100) substrate after the entire etching process using Ni film (1500 × 1500 µm^2^) with wet annealing at 1000 °C for 40 min. Diamond with a thickness of approximately 0.3 mm was completely pierced. The hole formed by piercing was also surrounded by four side walls.

### Flattening of a diamond (111) surface

Ni films were deposited on single-crystal diamond (111) substrates. Then, wet annealing at 900 °C for 60 min was conducted. Finally, the deposited films were removed using a hot mixed acid (HMA).

**Figure 5:**
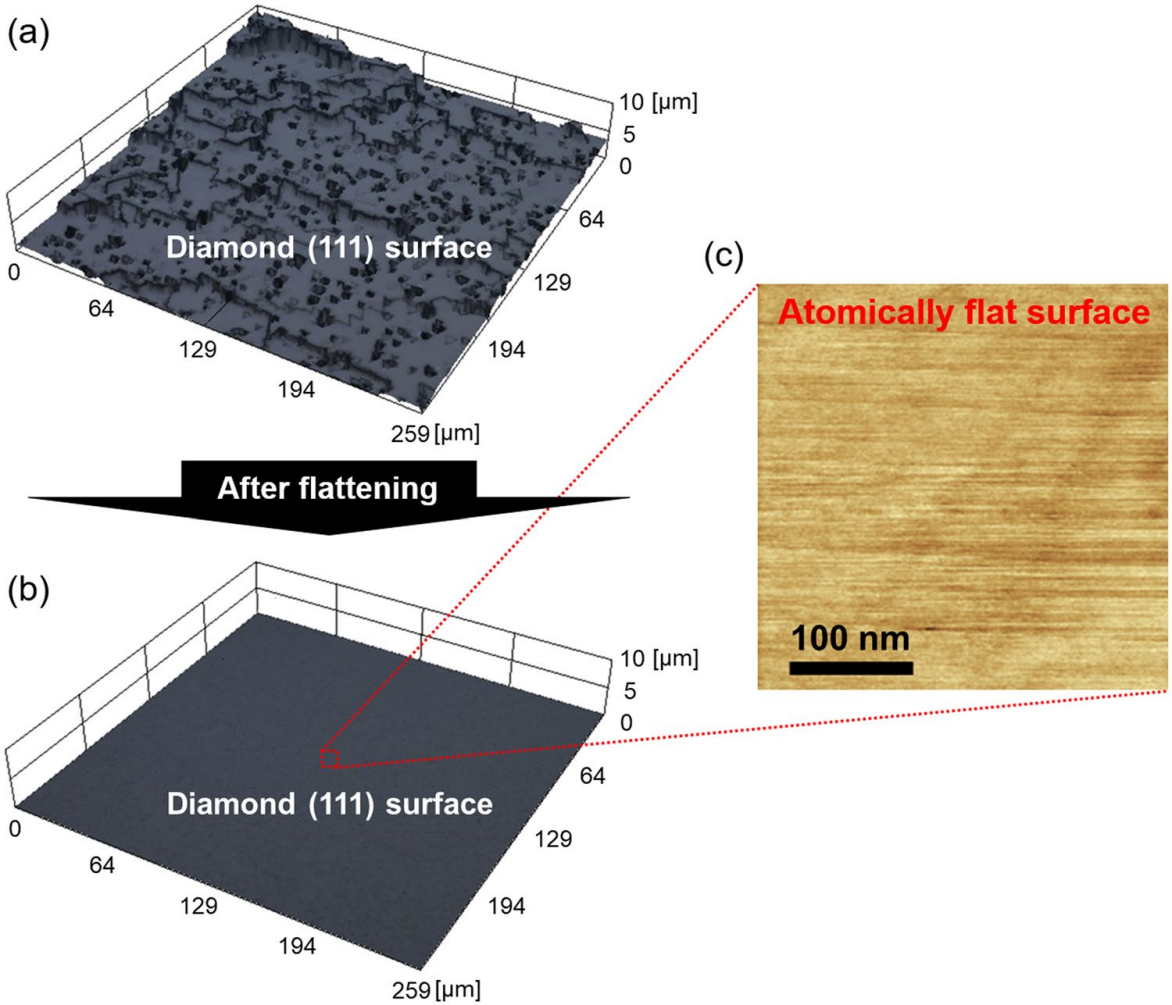
LM images of the diamond (111) surfaces before and after the proposed process with wet annealing at 900 °C for 60 min and an atomic force microscopy (AFM) image of the diamond (111) surface after the flattening.

Figure 5 shows the LM images of the diamond (111) surface before and after the process with wet annealing at 900 °C for 60 min and an atomic force microscopy (AFM) image of the diamond (111) surface after the process. Although the diamond (111) surface before the process exhibited a root-mean-square (RMS) roughness of 700 nm, the RMS roughness significantly decreased to less than the detection limit of the LM (~10 nm). According to the AFM measurements, the diamond (111) surface after the process exhibited considerably flat areas with an RMS roughness of less than or equal to 0.03 nm. These results indicate that diamond (111) is flattened through the process.

## Discussion

Diamond under the Ni films was etched with 46 µm-deep diamond trenches. This depth is sufficient for fabricating trench gate structures^2,37–39^. In contrast, as evidenced from the LM measurements, the thickness of the Ni-undeposited area on the diamond substrate did not change. Considering the vertical resolution of the LM, if any, the maximum etching depth at other locations could be less than approximately 10 nm. Therefore, a selectivity (diamond under Ni films/diamond at the Ni-undeposited area) of at least approximately 5000 is successfully obtained. The proposed process exhibited extremely higher selectivity than the plasma process with the maximum selectivity (diamond/mask) of 56^20^.

Diamond trenches were pointed or truncated inverted pyramids, which were surrounded by four side walls, and the angle between the side walls and (100) planes was approximately 55° (Fig. 1c). This angle is equivalent to that between the {111} planes and (100) planes. This indicates that the side walls of the diamond trenches are the {111} planes and they behave like etching stop planes. The anisotropy as mentioned above was extremely similar to that of Si etching by the KOH process^40,41^, which is frequently employed to fabricate Si trench structures for power devices. In reality, in terms of morphology, the diamond surface well-resembling the Si surface etched by the KOH process^40,41^ was obtained through the proposed diamond etching process (Fig. 2). This result strongly suggests the proposed process can be practically employed to form diamond trenches for power devices.

**Figure 6:**
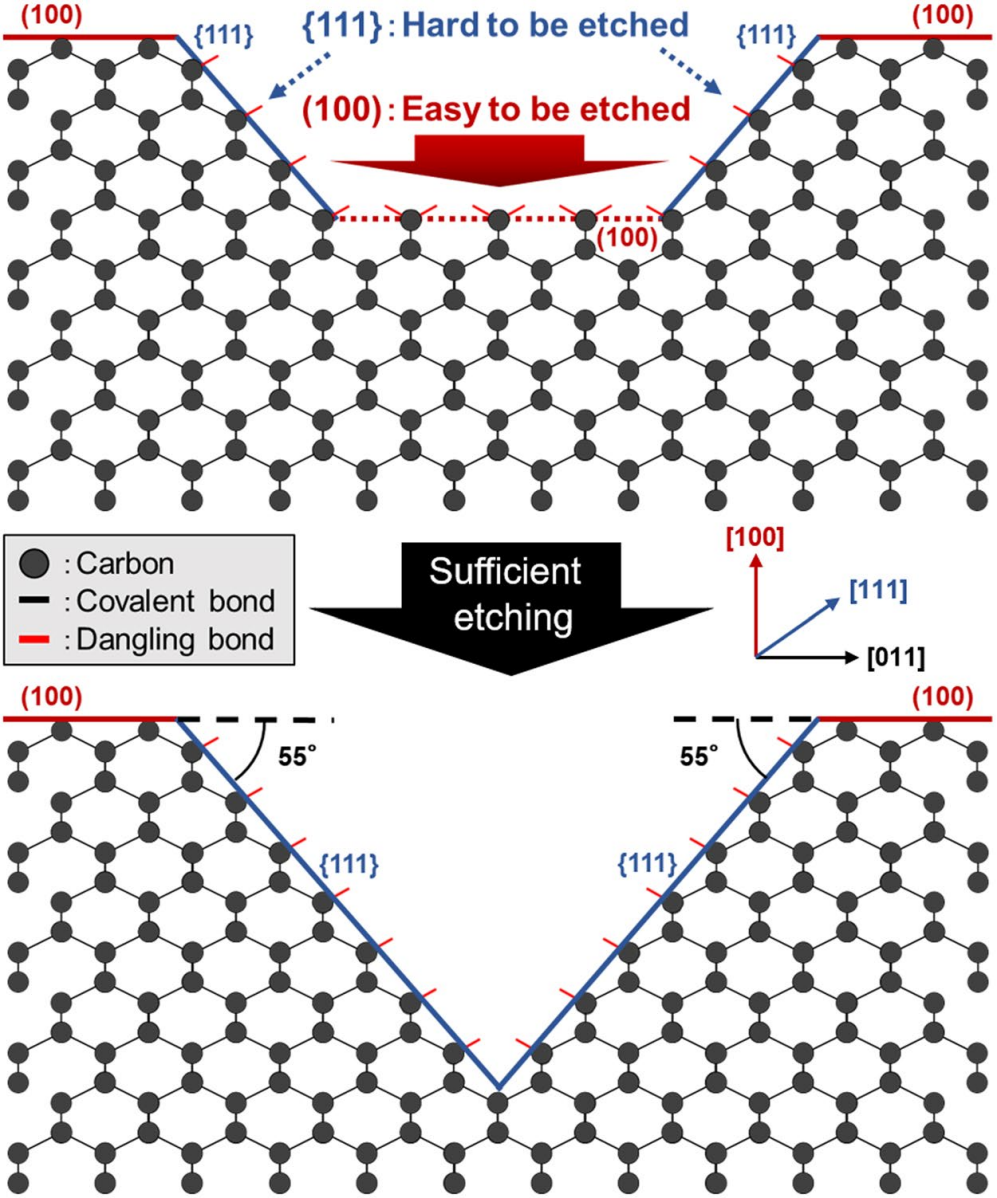
Schematic explaining the anisotropy of the proposed diamond etching based on the difference of the bonding power between C atoms comprising the (100) plane and those comprising the {111} planes.

One reason for the appearance of such anisotropy is supposedly related to that the diamond {111} planes constructed by carbon atoms with three covalent bonds are the most stable against the dissolution of carbon into Ni of all the orientation planes^42^ (Fig. 6). In fact, the severely rough diamond (111) surface with many steps (RMS roughness: ~700 nm), which was roughened by plasma treatments, was considerably flattened by the proposed process to the surface with an RMS roughness less than the detection limit of the LM (~10 nm). As ‘atomically flat’ corresponds to a surface roughness of less than or equal to 0.03 nm^43,44^, atomically flat diamond {111} surfaces can be obtained by etching diamond only at steps in the proposed process. The small size of the atomically flat surface possibly originates from the defects of diamond, contamination and insufficient processing time. Although further studies must be performed to elucidate the mechanisms for flattening the diamond surface and create considerably large atomically flat surfaces, the proposed process demonstrates an immense potential for replacing conventional polishing techniques.

The etching rates of the proposed process at 900, 950 and 1000 °C were estimated to be approximately 0.26, 2.3 and 8.7 µm/min, respectively. The etching rate of the proposed process at 900 °C was similar to that reported previously, i.e. 0.25 µm/min at 900 °C^27^. In contrast, the etching rate observed for the proposed process at 1000 °C was by far greater than those observed for other diamond-etching processes reported thus far, including plasma processes^27–30,45–51^. Owing to the exceedingly high rate and selectivity, piercing or cutting diamond, which is extremely difficult using other processes because of its low etching rate or selectivity, became considerably easier (Fig. 4).

**Figure 7:**
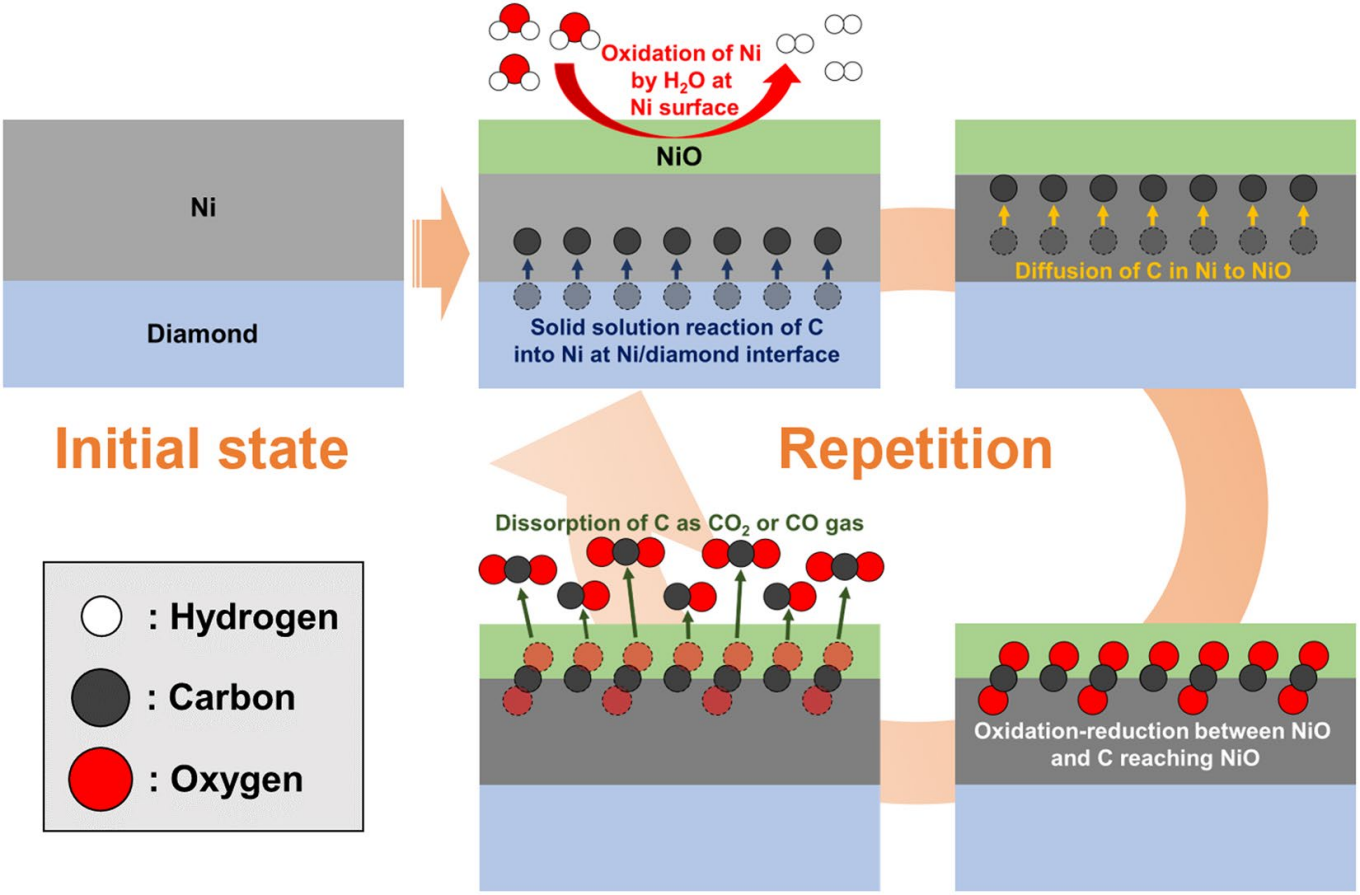
Schematics showing the mechanisms for the etching of diamond through the thermochemical reaction between Ni and diamond in high-temperature water vapour.

The possible mechanisms for the etching of diamond through the thermochemical reaction between Ni and diamond in high-temperature water vapour are shown in Fig. 7. First, C atoms on the diamond surface contacting the Ni film dissolve in the Ni film as a result of a solid solution reaction^27^. In addition, the Ni film surface is oxidised by water vapour^34^. Second, the dissolved C atoms diffuse towards NiO in accordance with the concentration gradient in the Ni film. Then, oxidation–reduction reaction between the NiO and C atoms reaching NiO occurs. Finally, the C atoms depriving the NiO of O desorb from the sample as CO_2_ and CO gases^52–55^. These steps are ceaselessly repeated during wet annealing. The discharge of C atoms prevents saturation of C atoms in Ni^28^. Thereby, the solid solution reaction is promoted, leading to high rate of diamond etching. The selective oxidation of Ni by water vapour is crucial for realising the continuous etching of diamond at a high rate.

## Methods

### Sample etching

The chemical-vapour-deposition-synthesised IIa-type single-crystal diamond (100) substrates, and the high-pressure- and high-temperature-synthesised (111) substrates with a surface roughened by plasma treatments were used. First, the substrates were immersed in H_2_SO_4_/H_2_O_2_ (1:2) at 120 °C for 15 min to remove surface contaminants. Second, Ni films with sizes of 30 × 30, 50 × 50, 100 × 100, 200 × 200 and 1500 × 1500 µm^2^ were deposited through metal masks onto the substrates by vacuum evaporation method. The thickness of the Ni films was approximately 0.2 µm. Third, the samples were annealed on a quartz plate in a quartz tube at 900 °C for 30, 60 and 120 min, at 950 °C for 5, 15 and 25 min, and at 1000 °C for 3, 5, 8, 13 and 40 min in water vapour using an electric furnace. The ramp-up rates of the temperature in the furnace was 20 °C/min from room temperature to 800 °C and 10 °C/min from 800 °C to 1000 °C. The water vapour was generated by bubbling N_2_ gas (400 sccm) through ultrapure water. This annealing is referred to as ‘wet annealing’. Finally, the samples were immersed in a HMA of H_2_SO_4_/HNO_3_ (3:1) at 220 °C for 20 min to remove the deposited films.

### Surface analysis

The surface morphology was observed via LM (LEXT OLS4100, Olympus), SEM (S-4500, Hitachi) and AFM (SPM-9700, Shimazu). The atomic composition and chemical bonding of the sample after wet annealing were investigated by XPS.
